# Predicting the 10-year risk of death from other causes in men with localized prostate cancer using patient-reported factors: Development of a tool

**DOI:** 10.1371/journal.pone.0240039

**Published:** 2020-12-07

**Authors:** Daniel M. Frendl, Gordon FitzGerald, Mara M. Epstein, Jeroan J. Allison, Mitchell H. Sokoloff, John E. Ware

**Affiliations:** 1 Department of Urology, Massachusetts General Hospital, Harvard Medical School, Boston, MA, United States of America; 2 Department of Quantitative Health Sciences, University of Massachusetts Medical School, Worcester, MA, United States of America; 3 Department of Surgery Center for Outcomes Research, University of Massachusetts Medical School, Worcester, MA, United States of America; 4 Department of Medicine, University of Massachusetts Medical School, Worcester, MA, United States of America; 5 Meyers Primary Care Institute, Worcester, MA, United States of America; 6 Department of Urology, University of Massachusetts Medical School, Worcester, MA, United States of America; 7 John Ware Research Group, Watertown, MA, United States of America; Universita degli Studi di Brescia, ITALY

## Abstract

**Objective:**

To develop a tool for estimating the 10-year risk of death from other causes in men with localized prostate cancer.

**Subjects and methods:**

We identified 2,425 patients from the Surveillance Epidemiology and End Results—Medicare Health Outcomes Survey database, age <80, newly diagnosed with clinical stage T1-T3a prostate cancer from 1/1/1998-12/31/2009, with follow-up through 2/28/2013. We developed a Fine and Gray competing-risks model for 10-year other cause mortality considering age, patient-reported comorbid medical conditions, component scores and items of the SF-36 Health Survey, activities of daily living, and sociodemographic characteristics. Model discrimination and calibration were compared to predictions from Social Security life table mortality risk estimates.

**Results:**

Over a median follow-up of 7.7 years, 76 men died of prostate-specific causes and 465 died of other causes. The strongest predictors of 10-year other cause mortality risk included increasing age at diagnosis, higher approximated Charlson Comorbidity Index score, worse patient-reported general health (fair or poor vs. excellent-good), smoking at diagnosis, and marital status (all other vs. married) (all p<0.05). Model discrimination improved over Social Security life tables (c-index of 0.70 vs. 0.59, respectively). Predictions were more accurate than predictions from the Social Security life tables, which overestimated risk in our population.

**Conclusions:**

We provide a tool for estimating the 10-year risk of dying from other causes when making decisions about treating prostate cancer using pre-treatment patient-reported characteristics.

## Introduction

Accurate assessment of life expectancy is critical to enabling evidence-based treatment decision-making for over 230,000 men diagnosed with prostate cancer in the U.S. annually [1]. Guidelines recommend that clinicians reserve definitive treatment of localized tumors for men with at least a 10-year life expectancy [2]. For men with localized low to intermediate risk tumors, the average risk of dying of prostate cancer in the 10 years after diagnosis is 5 to 8%, regardless of treatment [3]. In contrast, the risk of dying of causes other than prostate cancer (termed “other causes”) can range from as low as 6% to over 90%, depending on a man’s age, health, and social factors [3–6]. Thus, enabling accurate determinations of the risk of dying of other causes is of high importance, especially given that many men have historically been overtreated—dying of non-prostate causes within 10 years of treatment–while today a growing number of healthy older men, who may benefit from treatment, may not receive it because of their age alone [6–10].

Individualized mortality risk estimation is currently limited in real-world practice. The National Comprehensive Cancer Network guidelines recommend using risk estimates from the Social Security life tables, a method that relies on crude age-based life expectancy estimates [11,12]. Other available prediction tools, specifically developed for men with prostate cancer, incorporate both age and comorbidity for assessing other cause mortality risk [3,7,13,14]. There remains limited evidence supporting that these tools are meaningfully better than the Social Security life tables in assessing 10-year mortality risk [15]. Despite the availability of these tools, few clinicians use them in practice, most likely because they require busy clinicians’ valuable time to derive estimates [16]. Existing tools have also yet to evaluate whether integration of age and comorbidity information with patient-reported factors, such as physical functioning and general health, which are increasingly seen as valuable for comprehensively assessing health and other cause mortality risk, actually improve predictions [14,15,17,18]. Accurate mortality risk prediction is especially important for patients age 65–80, the age group most frequently receiving a new diagnosis of prostate cancer, who are more likely than younger men to die of other causes but still are often considered for definitive treatment. This study sought to identify a combination of pre-treatment patient-reported factors for estimation of the 10-year risk of death from other causes in men newly diagnosed with localized prostate cancer. We also sought to provide individualized risk predictions through a supplemental online risk estimation tool.

## Subjects and methods

The University of Massachusetts Medical School Institutional Review Board approved the study protocol. Given the data were analyzed anonymously using a large retrospective cohort consent was waived. IRB Review ID: H00002704.

### Data source

We used the Surveillance, Epidemiology, and End Results (SEER)—Medicare Health Outcomes Survey (MHOS) linked database that contains data from 22,023 men diagnosed with prostate cancer between 1998–2009, with follow-up through 2/28/2013. The SEER cancer registry collects sociodemographic, tumor, and treatment data representative of 26% of the U.S. population [19]. The Centers for Medicare & Medicaid Services’ (CMS) separately conducts the MHOS survey, annually collecting patient-reported comorbidity and health behavior data from a sample of Medicare Advantage Organization participants to monitor health outcomes, with response rates of 64–72% [20]. Medicare Advantage Organization participants represent roughly 19% of all Medicare beneficiaries. These independent SEER and MHOS databases have been linked with detailed methods previously reported elsewhere [20].

### Study population

Patients were selected if newly diagnosed with prostate cancer between 1/1/1998-12/31/2009, and completed an MHOS survey within the 3 years preceding their prostate cancer diagnosis. Exclusion criteria included diagnosis of prostate cancer on autopsy or death certificate or regionalized or metastasized tumors (stage >cT3a) and men over age 80 at diagnosis. We also excluded patients with other primary cancers diagnosed before prostate cancer. Age was restricted for the primary analysis because few men over age 80 have a life expectancy greater than 10 years and thus age alone may rule out definitive therapy [7]. Detailed exclusions are available in S1 Fig in S1 File.

### Data collected

Age at diagnosis was determined from the SEER registry. Patient-reported comorbidity data were available as (1) myocardial infarction; (2) congestive heart failure; (3) stroke; (4) emphysema, asthma or chronic obstructive pulmonary disease (COPD); (5) diabetes; (6) any cancer other than skin cancer; (7) paralysis or weakness on one side of the body; (8) Crohn’s disease, ulcerative colitis, or inflammatory bowel disease; (9) hypertension; (10) angina or coronary artery disease; (11) other heart conditions; (12) arthritis of the hip or knee; (13) arthritis of the hand or wrist; and (14) sciatica. An approximated Charlson Comorbidity Index (CCI) score was generated using Charlson weights for conditions 1–8, which overlap with the CCI [21].

Patient-reported physical and mental health-related quality of life were measured with the SF-36 Health Survey (v1) [22], administered to participants before 2006, and the VR-12 Health Survey [23], for men surveyed in 2006 and after. Physical Component Summary (PCS) and Mental Component Summary (MCS) scores were utilized as scored by MHOS with an algorithm that allows for comparing scores across SF-36 and VR-12 survey forms [24]. Scores are normalized to a mean of 50 points in the general U.S. population with a standard deviation of 10 points, with higher scores representing better health. Additionally, we used a single item, available in both surveys, assessing self-rated general health as excellent, very good, good, fair, and poor.

Data on activities of daily living were used as the modified Physical-Functioning Activities of Daily Living scale (PF-ADL), derived from 8 items available across all MHOS survey forms (moderate activities physical functioning item and ADLs: climbing stairs, bathing, dressing, eating, getting out of a chair, walking, toileting). This scale was Scores are reported on a 0–100 point scale, with higher scores representing better health.

Patients were considered smokers if reporting smoking every day or some days in MHOS. Individuals who SEER-MHOS categorized as “unknown smoking status” were considered non-smokers, as our exploratory analyses revealed they experienced survival similar to non-smokers. Education was categorized as “more than a high school education” or “high school education or less” to provide consistency with prior definitions [18]. Race-ethnicity was classified by SEER-MHOS as non-Hispanic white, non-Hispanic black, Hispanic, Asian/Pacific Islander, and other. MHOS variables for marital status (married vs. all other), home ownership, and household income were also assessed. Generally, the “unknown” or “missing” categories were treated as absence of exposure to the variable of interest. These categorizations enabled a complete case analysis that included 2,398 men.

### Survival and mortality outcomes

Survival was calculated as the difference between the date of diagnosis and the date of death, or last SEER follow-up date for censored patients. Patients not experiencing death within 10 years of prostate cancer diagnosis were censored at 10 years or at last SEER follow-up if followed <10 years. Patients who died were classified as having died of prostate-specific (PCSM) versus other causes, verified using the SEER cause of death recode variable. Patients missing follow-up data were excluded.

### Statistical analysis

Patient characteristics were compared among those dying of PCSM, OCM, and survivors using one-way analysis of variance tests and chi-squared tests as appropriate. Overall cumulative incidence of 10-year OCM was computed using a cumulative incidence function, a modified Kaplan-Meier method that accounts for the competing risk of dying of PCSM.

Fine and Gray competing risks models for 10-year OCM were fitted with PCSM as the competing event [25]. Pre-specified candidate predictors were selected based on prior predictive value reported in the literature. Candidate variables included age at diagnosis, comorbid medical conditions (approximated CCI), SF-36/VR12 PCS, SF-36/VR12 MCS, PF-ADL, self-rated general health, and sociodemographic variables (race, marital status, education level, household income) [3,13,14,18,26]. Akaike’s Information Criterion (AIC) and Harrell’s c-index (concordance index) guided model selection to optimize model parsimony and maximize discrimination [27]. Minimizing AIC limits the number of variables selected, penalizing more complex models that do not substantially fit the data better. Harrell’s c-index assesses a model’s ability to discriminate between individuals who live and die, ranging from poorest discrimination of 0.5 to perfect performance at 1.0 [28], and can be adapted to competing-risks models [29,30]. Interaction terms between age and comorbidity, comorbidity and general health, age and general health, and smoking and comorbidity were considered to evaluate potential effect modification. Risk scores were generated from the final model with the equation: risk of 10-year OCM = 1 –(baseline survival at 10 $\mathrm{years}{)}^{e^{\left(\sum x\hat{\beta }\right)}}$. Calibration of risk scores was assessed using a goodness of fit test comparing predicted OCM to observed cumulative incidence of OCM across quartiles of increasing predicted risk.

As a performance benchmark, the new model predictions were compared to the Social Security life tables [12–14]. Using the Social Security life tables annual risk of mortality, reported for the general male U.S. population [12], the risk of dying within the next 10 years of all-causes was computed and assigned to each patient with age rounded to integer values.

Although external data was not available for validating the model, we performed an internal validation of model discrimination using bootstrapped resampling methods. Briefly, to achieve an estimate of c-index optimism we re-estimated our model and discrimination in 100 bootstrapped samples. We then applied the re-estimated model predictions to the original complete data to also evaluate the discrimination of the new coefficient estimates in the original sample. Detailed methods are available in S1 Methods in S1 File. As an additional sensitivity analysis, the performance of the final model was evaluated in a sample including patients over age 80 who were excluded from the primary analysis. Subject characteristics and methods for this sensitivity analysis are described in supplementary materials. Additionally, SEER-MHOS death rates are lower than those observed in some populations, which may lead to systematic underestimation of mortality risk when applying the final risk estimates in other populations [7]. To improve the generalizability of the final risk model, exploratory re-calibrated model estimates are provided in S4 Table in S1 File. Detailed methods are available in S1 Methods in S1 File. All analyses were performed with Stata 12 (StataCorp, College Station, TX) or SAS 9.3 (SAS, Carry, NC) software, and the protocol is available online [31].

## Results

### Patient demographics

Mean age at diagnosis was 73 years. The majority of men (54%) had approximated CCI scores of 0, ranging from 0–7 points, with 21% of the sample having scores of 2 or higher. Compared to surviving men, those who died of OCM were older, with higher comorbidity burden, had worse PCS scores, worse general health, and were more likely to be smokers at diagnosis, and less often married. Overall, 67% of the cohort underwent definitive treatment for prostate cancer, the majority of which was radiation therapy. Notably, 56% of patients experiencing OCM within 10 years of diagnosis were treated aggressively- predominantly with radiation. Full sample characteristics are summarized in Table 1.

**Table 1:** Patient characteristics.

	Total	Prostate Specific Death	Other Cause Mortality	Surviving	*p*-value
**No. of patients (%)**	2425	76 (3)	465 (19)	1884 (78)	-
**Mean age at diagnosis**	72.9	74.5	73.8	72.6	<0.001
**Race (%)**					
Non-Hispanic White	1879 (77)	57 (75)	371 (80)	1451 (77)	0.282
Non-Hispanic Black	290 (12)	~	55 (12)	223 (12)	
Hispanic/Asian/Pacific Islander or other	256 (11)	~	39 (8)	210 (11)	
**Approximated Charlson Comorbidity Index Score (%)**					<0.001
0	1326 (54)	31 (40)	177 (38)	1118 (59)	
1	586 (24)	18 (24)	130 (28)	438 (23)	
≥2	513 (21)	27 (35)	158 (34)	328 (17)	
**Marital Status**					
Married vs. all other (%)	1695 (70)	48 (63)	292 (63)	1355 (72)	<0.001
**Smoker at diagnosis** (%)	301 (12)	18 (24)	86 (18)	197 (10)	<0.001
**Patient-reported Functioning and Wellbeing**					
PCS (mean, SD)	43.5 (10.9)	41.2 (11.6)	40.1 (11.1)	44.4 (10.4)	<0.001
MCS (mean, SD)	53.3 (9.4)	49.3 (10.7)	51.5 (10.4)	53.9 (8.9)	<0.001
Physical Functioning—ADL Index (mean, SD)^±^	87.8 (15.5)	83.1 (17.6)	83.4 (16.9)	89.2 (14.8)	<0.001
General Health (Fair or Poor) (n, %)	521 (22)	23 (30)	169 (37)	329 (18)	<0.001
**Tumor Grade (%)**					<0.001
Well to Moderately Differentiated	1630 (67)	30 (39)	338 (73)	1262 (67)	
Poorly Differentiated or Unavailable*	795 (33)	46 (61)	127 (27)	622 (33)	
**Tumor Clinical T-Stage (%)**					<0.001
cT1	1,193 (49)	20 (26)	227 (49)	939 (50)	
cT2	819 (34)	34 (45)	136 (29)	649 (34)	
T3a or Unavailable**	413 (17)	22 (29)	102 (22)	296 (16)	
**Primary Prostate Cancer Management (%)**					<0.001
Conservative	794 (33)	46 (60)	197 (42)	551 (29)	
Radical Prostatectomy	323 (13)	~	28 (6)	294 (16)	
Radiation Therapy	1308 (54)	~	240 (52)	1039 (55)	

~ cell size <11 individuals for some sub-categories, exact n =, % not reportable per SEER-MHOS data use agreements; the race variable was utilized as five levels in all analyses, including white, black, Hispanic, Asian-Pacific Islander, or other.

*The small percentage of ungraded tumors were not reported individually in this table due to cell size reporting limitations and represented <3% of the total sample.

**T3a tumors contributed less than 2% of the total sample; exact numbers were not reportable per SEER-MHOS data use agreements. Those without detailed T-stage available (<3% of the sample) did not have regionalized/metastasized tumors, as confirmed by other SEER staging variables.

^±^PF-ADL is scored on a 0–100 point scale with 0 being worse and 100 being best and is not normalized to a mean of 50 points.

### Fine and Gray competing risks regression analysis

Over a median follow-up of 7.7 years, 3.7% of the cohort died of PCSM and 24.3% of OCM. Median follow-up for survivors was 8.9 years (interquartile range 5.5–11 years). The combination of patient-reported pre-treatment factors with highest predictive value for death from OCM included age at diagnosis, approximated Charlson Comorbidity Index score, general health (excellent to good vs. fair or poor), smoking at time of diagnosis, and marital status (married vs. all other) (Table 2). The predictors are listed in the order of their respective contribution to the prediction, with age providing the greatest information and marital status providing the least. The model achieved a c-index of 0.70 vs. 0.59 from the Social Security life tables. The internal validation detailed in S1 Table in S1 File revealed the range of model discrimination results in the bootstrapped samples was 0.68 to 0.73 with a mean value of 0.70.

**Table 2:** Final adjusted model for the 10-year competing risk of other cause mortality.

Predictor	$\hat{\beta }$	Sub Hazard Ratio (SHR)	95% Confidence Interval	Z-Score	*p*-value for SHR	Harrell’s c-index**
**Age at diagnosis** (1-year increments)	0.078	**1.08**	1.06–1.11	6.44	<0.001	0.586
**Approximated Charlson Comorbidity Index Score**	0.197	**1.21**	1.14–1.30	6.04	<0.001	0.660
**Patient-reported general health**						0.678
Poor vs. (excellent/very good/good)	1.010	**2.75**	1.83–4.13	5.47	<0.001	
Fair vs. (excellent/very good/good)	0.623	**1.86**	1.49–2.33	4.84	<0.001	
**Smoker at diagnosis**	0.530	**1.70**	1.32–2.18	4.15	<0.001	0.693
**Marital status (all other vs. married)**	0.307	**1.36**	1.12–1.66	3.04	0.002	0.701

An online risk calculator supplement is available at http://www.urologyrisk.com

Baseline Survival: 0.99948996

10-Year Overall Cumulative Incidence of Non-Prostate Mortality: 24.307%

Model Harrell’s c-index: 0.701

Over the observation period, the total number of OCM deaths = 495 Total PCSM deaths = 76

**Harrell’s c-index is reported as the c-index for the model including the predictor of interest and any predictors listed above it. For example, the value reported for patient-reported general health is the c-index for a model with predictors: age, approximated Charlson Comorbidity Index Score, and the general health.

The final model with age, approximated CCI score, general health, smoking at diagnosis, and marital status performed as well as models that included more information about patient-reported physical and mental health (PCS and MCS) and models that included activities of daily living (PF-ADL) (c-index was 0.70 for all models with minimal differences in AIC). Other socioeconomic (education level, household income, homeownership) and race factors did not have significant associations with OCM in models that included age and approximated CCI score, except for being in the “other” race category, which did not have a clinically interpretable definition and represented a very few patients (4%). No significant interactions were observed, except for poor general health and an approximated CCI score of 7, which applied to <11 individuals and thus was left out of the final model. Model performance also remained similar in the sensitivity analysis that included patients over age 80 (S2 and S3 Tables in S1 File).

### Risk scores

Table 3 shows the range of risk scores obtained from the regression equation in this study population. Median predicted 10-year OCM risk was 20% (interquartile range 15–29%). The model was well calibrated in the SEER-MHOS population, with observed OCM mirroring predicted rates across increasing levels of risk (Table 4); in comparison, the Social Security life tables over-predicted risk for most patients in this study (Table 4). Example clinical scenarios where patients have sequentially poorer health and higher OCM risk in each example are available in S5 Table in S1 File. A dynamic risk calculation tool is available at http://www.urologyrisk.com.

**Table 3:** Range of 10-year other cause mortality risk predictions.

Risk Group	Predicted % 10-Year Non-Prostate Mortality Risk
Lowest 10%	12
Lowest 25%	15
Median Risk	20
Highest 75%	29
Highest 90%	42

An online risk calculator supplement is available at http://www.urologyrisk.com

Risk scores were calculated as 1-(base survival)^(exp(Σ$\hat{\beta }$x))

*SEER-MHOS risk estimates are derived from the model developed in the primary analysis (Table 2)

Base survival was 0.99948996 for the SEER-MHOS data calculations. $\Sigma \hat{\beta }x$ can be calculated as the sum of the ($\hat{\beta }$ presented in Table 2 times the value for the covariate).

**Table 4:** Model calibration and comparison to social security life table estimates: Predicted vs. observed other cause mortality.

Quartile of Risk Low to High	Mean SEER-MHOS Predicted % OCM (95% CI)	Observed Cumulative Incidence of OCM (95% CI)	Mean Social Security Life Table Predicted Mortality (95% CI)
1 (n = 599)	12.4 (12.3–12.6)	10.7 (8.0–13.9)	31.0 (30.5–31.4)
2 (n = 599)	17.6 (17.5–17.7)	20.4 (16.8–24.4)	38.8 (38.2–39.4)
3 (n = 599)	23.8 (23.6–24.0)	22.3 (18.5–26.3)	45.3 (44.5–46.1)
4 (n = 599)	43.4 (42.3–44.5)	45.5 (40.4–50.5)	45.3 (44.5–46.1)

## Discussion

This study developed a prognostic tool for 10-year other cause mortality risk in men newly diagnosed with prostate cancer. We identified a set of five pre-treatment patient-reported predictors that performed as well as more extensive and burdensome evaluations of health. While accurately predicting OCM risk for an individual patient remains challenging, our tool demonstrated improved discrimination between individuals who lived and died, as compared to estimates from currently recommended Social Security life tables [11]. Our tool also provided more accurate individualized estimates of mortality risk than Social Security life tables, which broadly overestimated risk for most patients in this study. These findings are particularly relevant as over half of patients experiencing OCM within 10 years of diagnosis were treated aggressively- predominantly with radiation, suggesting there may be room for improving our patient selection. Conversely, only 40% of those experiencing prostate-specific mortality underwent definitive treatment in this cohort of men over 65 –reinforcing the need to not simply rule out treatment for older individuals based on age alone.

The final predictors selected in this study were age, comorbidity, patient-reported general health, smoking, and marital status. These have all been individually shown to predict mortality risk, but our model is the first to combine these factors in a single prognostic calculator for 10-year OCM risk [3,7,13,14,18,26]. While age and comorbidity were the most important predictors, patient-reported general health, smoking, and marital status made valuable incremental contributions. Although we considered more detailed patient-reported assessments of health impairment in the modeling process, additional variables did not improve upon our final models’ ability to distinguish between those who lived and died.

There are a number of existing tools available for estimating geriatric life expectancy [32,33], as well as tools developed for estimating OCM risk in men with prostate cancer [3,7,13,14]. However, general geriatric tools require more extensive clinician inputs and are designed for shorter-term risk evaluation [32,33]. Further, there has been limited clinical adoption of existing tools for OCM risk estimation in men with prostate cancer [15,16]. Our tool improves upon Social Security life table risk estimates by providing more accurate individualized OCM risk estimates and better discrimination between those living and dying. We used reproducible patient-reported inputs and population-based data from men newly diagnosed with prostate cancer to promote the generalizability of our predictions. We facilitate use by providing a supplemental online interface (http://www.urologyrisk.com). Risk scores can be interpreted relative to the population risk distribution (provided in Table 3), rather than as absolute values alone. For example, clinicians may consider focusing detailed discussions of definitive therapies on patients who fall in the lower risk quartiles, who have a low risk of dying within 10 years of diagnosis.

This work has several strengths and limitations. The generalizability of our findings may be limited as MHOS surveys Medicare Advantage plan enrollees who, on average, have better self-reported health and lower comorbidity burden than men enrolled in traditional fee-for service Medicare [34]. This may mean that our model could possibly underestimate the absolute OCM risk when applied to the general population of men over 65. However, prior work has shown that, after appropriately adjusting for comorbidity burden and health status, outcomes are comparable between Medicare Advantage and fee-for service patients [34]. We would caution that external validation of our model is needed before broad utilization of our risk estimates. Regarding comorbidities, while we captured the majority of conditions listed in the Charlson Comorbidity index, we were unable to account for three conditions: end-stage renal disease, liver failure, and peptic ulcer disease, which were not recorded in SEER-MHOS. This may have contributed to our model’s overall modest utility for discrimination given a c-index of 0.70 [17]. Furthermore, we would like to emphasize that if risk estimates from our prediction model are applied to external data it is quite likely that the model may not discriminate as well as it has in this report. While our internal validation methods suggested minimal optimism bias in our c-index estimates, no internal validation can substitute for a robust validation in external data. However, in comparison, the discrimination of the recommended Social Security life tables would be considered of no clinical value (c-index 0.50–0.60) [17]. While our model discrimination is overall modest, it is important to also consider that a third to a half of patients with prostate cancer die of events that may be difficult to predict (e.g., subsequent development of other cancers, acute cardiac events and stroke) [18]. Thus, there may be inherent limitations to how well any prognostic model can predict who will live and die within 10-years until better disease markers are available that predict events that are currently difficult to forecast. Additionally, we have opted not to incorporate definitive treatment decisions into our model. This may lead our model to overestimate OCM risk among men who clinicians would subjectively deem good candidates for definitive treatment and underestimate OCM risk among men who clinicians would subjectively consider poor candidates for intervention. Finally, because a high proportion of our population underwent initial definitive treatment, our population may overall reflect a healthier cohort, which may lead our model to underestimate absolute OCM risk in cohorts of men who are subjectively poor candidates for intervention.

## Conclusions

This study provides a prognostic tool which uses a simple set of patient-reported characteristics that may better identify men at high and low risk of 10-year non-prostate cancer mortality than Social Security life tables.

## Supporting information

S1 File (DOCX)
