# Supplementary material for: Predicting the 10-year risk of death from other causes in men with localized prostate cancer using patient-reported factors: Development of a tool
Source: PLoS One. 2020 Dec 7;15(12):e0240039. doi: 10.1371/journal.pone.0240039 (PMC7721137; doi:10.1371/journal.pone.0240039)
Supplement: S1 File — (DOCX) [file pone.0240039.s001.docx]

**S1 Methods:**

**Internal Validation Methods:**

We refitted the final regression model using bootstrapped resampling methods to perform an internal validation of the model’s discrimination. We opted to use bootstrapped resampling over split-sample validation methods given the relatively small sample size and potential power limitations with sample splitting. In the bootstrapped samples we refit the regression model and estimated discrimination statistics. We also applied risk estimates from the new regression coefficient estimates back in the original cohort, again recalculating discrimination. This procedure was performed with 100 bootstrapped samples. The range of c-index results in the bootstrapped samples was 0.68-0.73 with a mean value of 0.70. Table S1 summarizes the results of this internal validation. Despite the consistent discrimination statistics obtained with our internal validation process, we believe it is likely that our model discrimination may be worse in external samples. We believe internal validation methods are limited in their ability to provide insight into the model’s performance in external populations. Therefore, we would again emphasize that if risk estimates from our prediction model are applied to external data it is quite likely that the model may not discriminate as well as it has in this report. Future external validation will be needed.

**Analysis including patients over age 80:**

Additionally, we refitted the regression in all patients age 66 and older at prostate cancer diagnosis to assess the performance of risk estimates in a population that included the oldest patients. Patient characteristics are detailed in Table S2. The final model identified in the primary sample was refitted in this older sample to evaluate whether variables performed differently when patients over 80 were included in the sample and to assess to what extent model discrimination changed by including individuals with a high probability of death. The performance of the benchmark models was also re-assessed in this population. Results of these analyses are reported in Table S3.

**Exploratory Recalibration:**

Because SEER-MHOS death rates are lower than those observed in some populations this may lead to systematic underestimation of mortality risk when applying the final risk estimates in other populations.^1^ To provide an estimate of the range of risk scores our model may estimate when refitted in external data, exploratory re-calibrated model estimates are provided in Table S4. This was achieved by adjusting the baseline survival rate in the final risk equation presented above to achieve a mean 10-year other cause mortality rate of 36.9%, obtained by applying age-matched 10-year all-cause mortality risk estimates (39.9%) in this population from the Social Security life tables, minus 3% to account for prostate-specific mortality.^2^

**References:**

1. Daskivich TJ, Lai J, Dick AW, et al. Variation in treatment associated with life expectancy in a population-based cohort of men with early-stage prostate cancer. *Cancer*. July 2014:1-9.

2. Social Security Administration Actuarial Life Table.

**S1 Table: Internal validation: Re-estimation of model discrimination in 100 bootstrap resampled datasets and recalculated discrimination using the new regression risk estimates applied in the original cohort.**

| **Run #** | **C-index of re-estimated model in resampled data** | **C-index estimated in original data with new beta-hat estimates** |
| --- | --- | --- |
| Original Full Model Cohort: | NA | 0.700 |
| Bootstrap Resample Mean C-index: | 0.703 | 0.699 |
| Standard Deviation of C-index values: | 0.012 | 0.001 |
| Bootstrap Resample Minimum C-index: | 0.686 | 0.697 |
| Bootstrap Resample Maximum C-index: | 0.730 | 0.702 |

**S2 Table: Patient characteristics secondary analysis (age 66+ group)**

|  | **Total** | **Prostate Specific Death** | **Other Cause Mortality** | **Surviving** | ***p*-value** |
| --- | --- | --- | --- | --- | --- |
| **No. of patients (%)** | 2854 | 116 (4) | 648 (23) | 2090 (73) | - |
| **Mean age at diagnosis** | 75.1 | 78.8 | 77.3 | 74.3 | <0.001 |
| **No. race (%)** |  |  |  |  |  |
| Non-Hispanic White | 2221 (78) | 90 (78) | 524 (81) | 1607 (77) | 0.09 |
| Non-Hispanic Black | 311 (11) | 13 (11) | 66 (10) | 232 (11) |  |
| Hispanic/Asian/Pacific Islander or other | 322 (11) | 13 (11) | 58 (9) | 251 (12) |  |
| **Approximated Charlson Comorbidity Index Score (%)** |  |  |  |  | <0.001 |
| 0 | 1582 (55) | 50 (43) | 259 (40) | 1273 (61) |  |
| 1 | 674 (24) | 27 (23) | 172 (27) | 475 (23) |  |
| ≥2 | 598 (21) | 39 (34) | 217 (33) | 342 (16) |  |
| **Marital Status** |  |  |  |  |  |
| Married vs. all other (%) | 1948 (68) | 69 (59) | 390 (60) | 1489 (71) | <0.001 |
| **Smoker at diagnosis** (%) | 301 (12) | 18 (24) | 86 (18) | 197 (10) | <0.001 |
| **Patient Reported Functioning and Wellbeing** |  |  |  |  |  |
| PCS (mean, SD) | 43.5 (10.9) | 41.2 (11.6) | 40.1 (11.1) | 44.4 (10.4) | <0.001 |
| MCS (mean, SD) | 53.3 (9.4) | 49.3 (10.7) | 51.5 (10.4) | 53.9 (8.9) | <0.001 |
| Physical Functioning - ADL Index (mean, SD)^±^ | 87.8 (15.5) | 83.1 (17.6) | 83.4 (16.9) | 89.2 (14.8) | <0.001 |
| General Health (Fair or Poor) (n, %) | 521 (22) | 23 (30) | 169 (37) | 329 (18) | <0.001 |
| **Tumor Grade** |  |  |  |  | <0.001 |
| Well to Moderately Differentiated | 1833 (64) | 30 (39) | 338 (73) | 1351 (65) |  |
| Poorly Differentiated or Unavailable* | 1021 (36) | 46 (61) | 127 (27) | 739 (35) |  |
| **Tumor Clinical T-Stage** |  |  |  |  | <0.001 |
| cT1 | 1,372 (48) | 37 (32) | 302 (47) | 1033 (49) |  |
| cT2 | 981 (34) | 46 (39) | 191 (29) | 744 (36) |  |
| c T3a or Unavailable** | 501 (18) | 33 (28) | 155 (24) | 313 (15) |  |
| **Prostate Cancer Management** |  |  |  |  |  |
| Conservative | 1148 (40) | 83 (71) | 356 (54) | 709 (34) | <0.001 |
| Radical Prostatectomy | 300 (11) | ~ | 26 (4) | 273 (13) |  |
| Radiation Therapy | 1406 (49) | ~ | 266 (41) | 1108 (53) |  |
| ~ cell size <11 individuals for some sub-categories, exact n=,% not reportable per SEER-MHOS data use agreements; the race variable was utilized as five levels in all analyses, including white, black, Hispanic, Asian-Pacific Islander, or other.  Mean follow-up time for this cohort was 7.2 years  *The small % of ungraded tumors were not reported individually in this table due to reporting limitations and represented <3% of the total sample.  **T3a tumors contributed less than 2% of the total sample; exact numbers were not reportable per SEER-MHOS data use agreements. Those without detailed T-stage available (<3% of the sample) did not have regionalized/metastasized tumors, as confirmed by other SEER staging variables.  ^±^PF-ADL is scored on a 0-100 point scale with 0 being worse and 100 being best and is not normalized to a mean of 50 points | | | | | |

**S3 Table: Final Adjusted Fine and Gray Proportional Hazards Model for 10-Year Competing Risk of Other Cause Mortality in men >66 years of age**

| **Predictor** | $\hat{\beta}$ | **Adjusted Sub Hazard Ratio (SHR)** | **95% Confidence Interval** | **Z-Score** | ***p*-value for Adjusted SHR** |
| --- | --- | --- | --- | --- | --- |
| **Age at diagnosis** (1 year increments) | 0.079 | **1.08** | 1.07-1.10 | 10.93 | <0.001 |
| **Approximated Charlson Comorbidity Index Score** | 0.199 | **1.22** | 1.15-1.29 | 6.81 | <0.001 |
| **Patient-reported general health** |  |  |  |  |  |
| Poor vs. (excellent/very good/good) | 0.709 | **2.03** | 1.37-3.00 | 3.55 | <0.001 |
| Fair vs. (excellent/very good/good) | 0.465 | **1.59** | 1.32-1.92 | 4.81 | <0.001 |
| **Smoker at diagnosis** | 0.536 | **1.71** | 1.37-2.13 | 4.78 | <0.001 |
| **Marital status (all other vs. married)** | 0.275 | **1.32** | 1.11-1.56 | 3.20 | 0.001 |
| *Baseline Survival*: 0.99947333  *10-Year Overall Cumulative Incidence of Non-Prostate Mortality*: 26.101%  *Model Harrell’s c-index: 0.72; Social Security life tables c-index: 0.65; Other comparator nomogram c-index values: 0.68 and 0.69* | | | | | |

**S4 Table: Recalibrated Range of 10-Year Other Cause Mortality Risk Predictions**

|  | **Predicted % 10-Year**  **Non-Prostate Mortality Risk** | |  |
| --- | --- | --- | --- |
| **Risk Group** | **SEER-MHOS Predicted*** | **Exploratory Recalibrated High Estimate*** |  |
| Lowest 10% | 12 | 20 |  |
| Lowest 25% | 15 | 25 |  |
| Median Risk | 20 | 33 |  |
| Highest 75% | 29 | 44 |  |
| Highest 90% | 42 | 61 |  |
| *An online risk calculator supplement is available at* [*http://www.urologyrisk.com*](http://www.urologyrisk.com)  Risk scores were calculated as 1-(base survival)^(exp(Σ$\hat{\beta}$x))  *SEER-MHOS risk estimates are derived from the model developed in the primary analysis (Table 2); exploratory recalibrated high estimates represent exploratory predictions from the recalibrated model.  Base survival was 0.99948996 for the SEER-MHOS data calculations and 0.999115237 for the recalibrated estimates. Σ$\hat{\beta}$x can be calculated as the sum of the ($\hat{\beta}$ presented in table 2 times the value for the covariate). | | | |

**S5 Table: Sample Clinical Scenarios with increasing 10-Year Other Cause Mortality Risk**

|  |  |  | **Predicted 10-Year Other Cause Mortality Risk (%)** | |  |
| --- | --- | --- | --- | --- | --- |
| **Case Risk** | **Case Description** | **Age at Diagnosis** | **SEER-MHOS Risk Estimate*** | **Exploratory Recalibrated High Estimate*** |  |
| Lowest | A non-smoker who is married, rates health as “good”  with CCI score of 0 | 65 | 8 | 13 |  |
|  |  | 70 | 12 | 19 |  |
|  |  | 75 | 17 | 27 |  |
|  |  | 80 | 24 | 37 |  |
| Lower | A non-smoker who is married, rates health as “good”,  with CCI score of 1 | 65 | 10 | 16 |  |
|  |  | 70 | 14 | 23 |  |
|  |  | 75 | 20 | 32 |  |
|  |  | 80 | 28 | 43 |  |
| Moderate | Smoker who is married, rates heath as “fair”,  with CCI score of 1 | 65 | 25 | 43 |  |
|  |  | 70 | 38 | 56 |  |
|  |  | 75 | 50 | 70 |  |
|  |  | 80 | 65 | 84 |  |
| Moderate High | Smoker who is married, rates health as “poor”,  with CCI score of 2 | 65 | 44 | 63 |  |
|  |  | 70 | 57 | 77 |  |
|  |  | 75 | 72 | 89 |  |
|  |  | 80 | 85 | 96 |  |
| High | Smoker who is unmarried, rates health as “poor”,  with a CCI score of 3 | 65 | 61 | 81 |  |
|  |  | 70 | 76 | 91 |  |
|  |  | 75 | 88 | 97 |  |
|  |  | 80 | 95 | 99 |  |
| *SEER-MHOS risk estimates are derived from the model developed in the primary analysis (Table 2); exploratory high range estimates represent exploratory predictions from recalibrated model.  Risk scores were calculated as 1-(base survival)^(exp(Σ$\hat{\beta}$x))  Base survival was 0.99948996 for the SEER-MHOS data calculations and 0.999115237 for the recalibrated estimates. Σ$\hat{\beta}$x can be calculated as the sum of the ($\hat{\beta}$ presented in Table 4.2 times the value for the covariate).  CCI= approximated Charlson Comorbidity Index | | | | | |

**S1 Fig: Study Cohort Selection Flow Diagram**


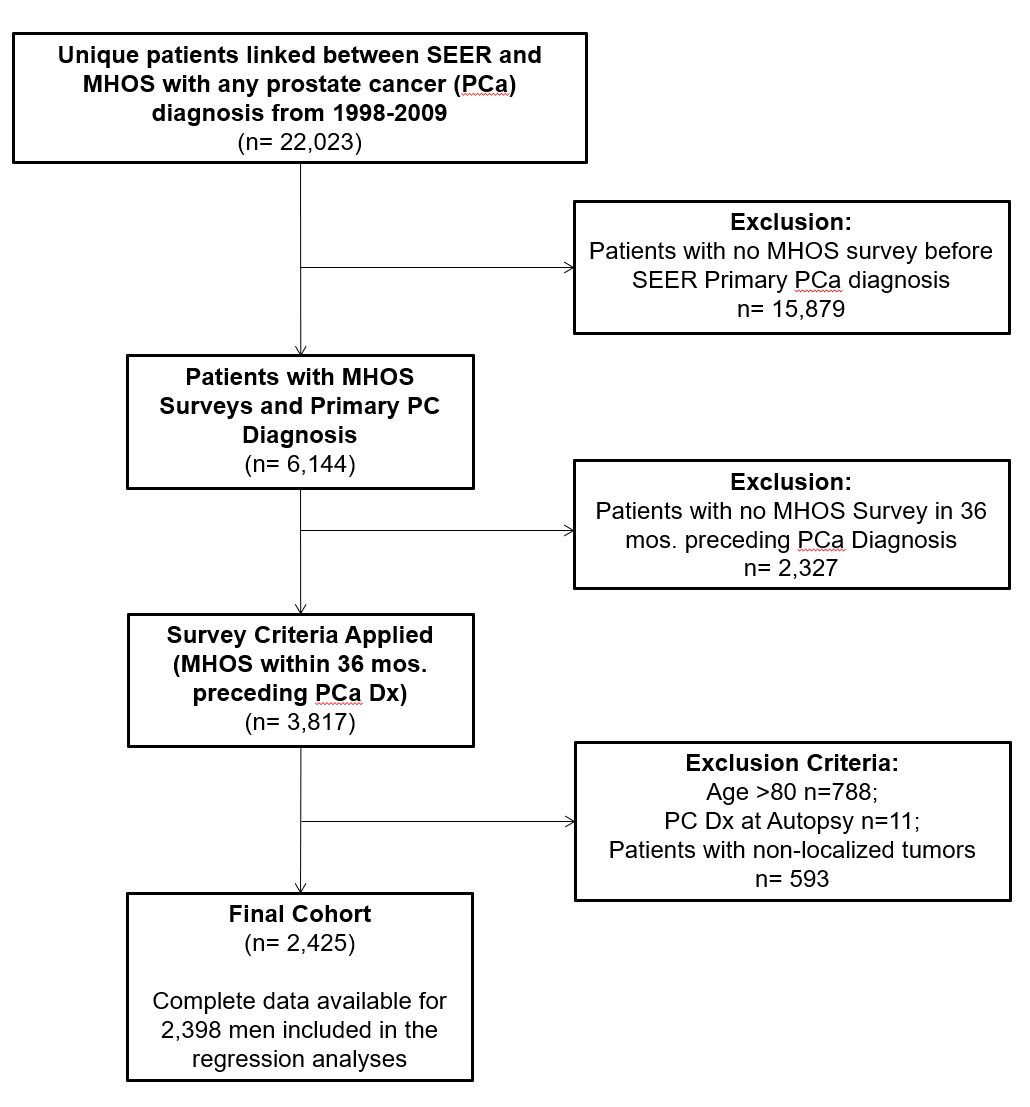


**S2 Fig: Calibration of the final model in the Surveillance Epidemiology and End Results – Medicare Health Outcomes Survey data set.** The diagonal line indicates perfect calibration.
